# Monogenetic epilepsies and how to approach them in 2022

**DOI:** 10.1515/medgen-2022-2143

**Published:** 2022-09-22

**Authors:** Ilona Krey, Konrad Platzer, Johannes R. Lemke

**Affiliations:** Institute of Human Genetics, University of Leipzig Medical Center, Leipzig, Germany; Center for Rare Diseases, University of Leipzig Medical Center, Leipzig, Germany

## Introduction

Almost 50 million people worldwide, or nearly 1 % of the general population, develop epilepsy during their lifetime [3]. There are two age peaks: early childhood and late adulthood [5]. Hence, very different causalities contribute to epileptogenesis depending on the age of manifestation. In late adulthood, cerebrovascular events and neoplasia are the major conditions triggering new onset of epilepsy, which will as a consequence usually be of focal origin. In early childhood, however, congenital – and thus genetic – causes predominate, which have a high likelihood of being of generalized origin.

Clinical classification of the epilepsies depends on various aspects, such as age at onset, seizure types, electroencephalogram (EEG), and magnet resonance imaging (MRI) [18]. Three main groups can be distinguished:

– **Focal epilepsies** (FE) are characterized by epileptic activity on the EEG arising from a specific part (e. g. a particular lobe) of the brain. FE can manifest with focal seizures, though secondary generalization, generalized seizures may just as well occur. FE accounts for ∼60 % of all epilepsies [6].
– **Generalized epilepsies** (GE) are characterized by epileptic activity on the EEG affecting not just a particular focus but the whole or large parts of the brain. GE are associated with a broad spectrum of generalized seizures, including absence, myoclonic, tonic-clonic, atonic, and many other seizures. GE accounts for ∼40 % of all epilepsies [6].
– **Developmental and epileptic encephalopathies** (DEE) refer to a group of disorders characterized by both developmental delay or even regression and epilepsy. Seizures may be diverse and often drug-resistant and may sometimes be associated with recognizable EEG patterns [18]. DEE are individually very rare and the overall annual incidence of monogenic forms is estimated to be around 1 per 2.100 live births [21].

Despite the existence of a few recognizable disorders with isolated epilepsy as the core phenotype, the most important feature pointing towards a potential monogenic aetiology of any epilepsy is the presence of associated co-morbidities, first and foremost of developmental delay and/or intellectual disability. However, this aspect is not well addressed in clinical classification schemes [18] and thus these approaches appear ineligible to depict the vast aetiologic heterogeneity of epileptic disorders.

The present article aims (i) to provide an overview on what is known on the genetic background of epileptic disorders in general, (ii) to summarize the experience in using various genetic testing approaches, and (iii) to recommend a useful and pragmatic approach for identifying genetic causes in individuals with seemingly unclassified epilepsy disorders.

## Genetic background of the epilepsies

**FE:** Despite dramatic advances in high-resolution MRI, the morphological cause of FE remains unclear in the majority of cases and only a miniscule fraction of these cases can be assigned to known monogenic entities.

The most common form of FE is temporal lobe epilepsy (TLE), where seizures are the single core feature and neurodevelopmental co-morbidities are usually not present. Only four genes have currently been linked to very rare familiar forms of TLE (Table 1) but these do not appear to play any role in singular TLE cases.

Genetic testing reveals a diagnostic yield of around 1 % in FE (including TLE) [8], [23] and appears to be negligible if the individual phenotype does not point towards a specific (mostly familial) entity. The aetiology of FE is generally considered to be more complex.

Individuals with FE due to focal cortical dysplasia occasionally exhibit somatic variants in genes of the *mechanistic target of rapamycin* (mTOR) pathway (Table 2) [1], [9], [13], [19]. The detection of these somatic mosaics goes beyond routine genetic testing and requires analysis of the affected tissue (especially of the brain, e. g. after epilepsy surgery) rather than blood [8].

**Table 1:** Genes associated with familial focal epilepsy (germline variants).

Gene	Phenotype
*CPA6*	Familial temporal lobe epilepsy
*GAL*	Familial temporal lobe epilepsy
*LGI1*	Familial temporal lobe epilepsy
*RELN*	Familial temporal lobe epilepsy
*CHRNA2*	Nocturnal frontal lobe epilepsy
*CHRNA4*	Nocturnal frontal lobe epilepsy
*CHRNB2*	Nocturnal frontal lobe epilepsy
*KCNT1*	Nocturnal frontal lobe epilepsy
*DEPDC5*	Familial focal epilepsy with variable foci
*NPRL2*	Familial focal epilepsy with variable foci
*NPRL3*	Familial focal epilepsy with variable foci
*SCN3A*	Familial focal epilepsy with variable foci
*GRIN2A*	Focal epilepsy with centrotemporal (Rolandic) spikes

**Table 2:** Genes associated with single cases of focal epilepsy (somatic variants).

Gene	Phenotype
*PIK3CA*	Focal cortical dysplasia type II / hemimegalencephaly
*AKT3*	Focal cortical dysplasia type II / hemimegalencephaly
*TSC1*	Focal cortical dysplasia type II / hemimegalencephaly
*TSC2*	Focal cortical dysplasia type II / hemimegalencephaly
*RHEB*	Focal cortical dysplasia type II / hemimegalencephaly
*MTOR*	Focal cortical dysplasia type II / hemimegalencephaly
*DEPDC5*	Focal cortical dysplasia type II / hemimegalencephaly
*SLC35A2*	Focal cortical dysplasia type I / malformation of cortical development

**GE:** Due to numerous reports on large pedigrees, GE is considered to be of genetic origin and even has officially been named “genetic generalized epilepsy” (GGE) [18].

The most common forms of (G)GE comprise childhood absence epilepsy, juvenile myoclonic epilepsy, and GE with febrile seizures plus. Familial aggregation and co-occurrence are frequently seen among these disorders. However, despite the identification of a few genes (Table 3) in rare familial cases with often more complex phenotypes comprising neurodevelopmental co-morbidities, the genetic cause of single individuals with isolated (G)GE usually remains unclear.

Genetic testing reveals a diagnostic yield of up to 3 % in (G)GE [14] and appears to play a very minor role in diagnostics of isolated cases with non-syndromic phenotypes. However, in case of co-occurring neurodevelopmental issues, the potential diagnostic yield can increase considerably and syndromal types of GE can be found in various microdeletion syndromes (i. e. 15q11.2, 15q13.3, 16p13.11, 22q11.2) as well as several types of monogenic DEE.

**Table 3:** Genes associated with genetic generalized epilepsy.

Gene	Phenotype
*SCN1A*	Generalized epilepsy with febrile seizures plus
*SCN1B*	Generalized epilepsy with febrile seizures plus
*GABRD*	Idiopathic generalized epilepsy
*GABRG2*	Generalized epilepsy with febrile seizures plus
*HCN1*	Generalized epilepsy with febrile seizures plus
*HCN2*	Generalized epilepsy with febrile seizures plus
*STX1B*	Generalized epilepsy with febrile seizures plus
*SLC2A1*	GLUT1 deficiency

**DEE:** Despite being the least prevalent sub-group of epilepsy disorders, DEE are particularly likely to be of monogenic origin.

According to OMIM (https://www.omim.org/phenotypicSeries/PS308350), more than 100 genes have been associated with different types of DEE. As DEE phenotypes can be disturbingly unspecific and enormously overlapping, a clinical categorization will only be able to capture a tiny fraction of cases and only those with the most unambiguous phenotypic features. Therefore, clinical classification approaches of DEE do not reflect its exceedingly heterogeneous genetic aetiology.

Genetic testing reveals a diagnostic yield of up to 60 % in DEE, particularly in neonatal or early-infantile DEE, [15], [16], [22] and is therefore a very powerful tool in establishing an aetiologic diagnosis in an affected individual.

## Genetic testing of the epilepsies

In the past years, the possibilities of identifying disease-causing genetic alterations have improved significantly. This improvement was not only due to technical advances but also an impressive increase of genetic databases with respect to both disease-associated variants and healthy controls. These attainments allow for a reconsideration of conventional and even recent genetic testing approaches in routine diagnostic settings.

**Exome sequencing** is currently the method of choice in genetic testing of DEE as it does reflect the vast heterogeneity of this disease spectrum and is associated with a remarkably high diagnostic yield of up to 45 % [17] (or higher in early-onset DEE). It is therefore recommended as first-tier diagnostics in DEE by the International League Against Epilepsy (ILAE) [11]. Analysis should implicitly include evaluation of both single nucleotide variants (SNVs) and copy number variants (CNVs) and should preferably be performed in the setting of parent–offspring trio sequencing [11].

**Panel sequencing** enables a diagnostic yield of ∼25 % in DEE [11], [12], [22] (or higher in early-onset DEE [15], [16], [22]), which is considerably less compared to exome sequencing. There are currently no standards or recommendations regarding design, size, or quality of a gene panel. Therefore, all these parameters vary between each individual provider and the common overlap is disturbingly low [7]. Moreover, panel designs are quickly outdated given the still steadily growing number of newly recognized DEE genes. And last but not least, the targeted enrichment of a limited number of genes prevents a comprehensive CNV analysis. Panel sequencing should therefore not be part of routine diagnostics of DEE and should only be used if exome sequencing is not available. However, it may be considered as a virtual panel approach on the basis of an exome sequencing dataset.

**Genome sequencing** will likely succeed and replace exome sequencing within the next few years. Its current diagnostic yield in DEE is 48 % [20], which is only marginally higher compared to exome sequencing. Still, genome sequencing requires more elaborate and costly investments in laboratory equipment as well as bioinformatic pipelines. Thus, it currently remains a challenging task to perform genome sequencing just as cost-efficiently as exome sequencing.

**Sanger sequencing** had been the golden standard for molecular genetic sequencing for many years. However, it appears to be more vulnerable to sequencing errors than previously thought [4]. Moreover, Sanger sequencing cannot compete with high-throughput sequencing techniques regarding diagnostic yield as well as cost efficiency. This holds true even for types of DEE with relatively low genetic heterogeneity, such as Dravet syndrome. Sanger sequencing should therefore not be part of routine diagnostics of DEE and should only be considered for validation or targeted familial segregation analyses.

**Chromosomal microarrays** (CMAs) have revolutionized diagnostics of DEE many years ago and enabled a relevant diagnostic yield of up to 16 % [2]. However, their resolution cannot compete with that of contemporary CNV analysis based on exome sequencing data. CMA should therefore not be part of routine diagnostics of DEE and should only be considered for validation analyses.

**Methylation-specific multiplex ligation-dependent probe amplification** (MS-MLPA) is a molecular genetic testing method that should only be considered if a respective diagnosis (e. g. Angelman syndrome, Prader–Willi syndrome, etc.) is specifically being suspected. MS-MLPA is not a useful screening method within standard routine diagnostics of DEE.

**Fragment length analysis** should only be considered if a respective diagnosis (e. g. Fragile-X syndrome, etc.) is specifically being suspected. It is not a useful screening method within standard routine diagnostics of DEE [24].

**Chromosomal karyotyping** has for a very long time been the initial component of a routine diagnostic work-up of individuals with DEE and developmental disorders in general. However, the diagnostic yield is remarkably low [24] and basically all potentially detectable aberrations can also be detected by more contemporary CNV analysis [24]. A sole example of chromosomal DEE that may potentially escape CNV analysis are ring chromosomes, e. g. ring 20. As such diagnoses are extraordinarily rare, chromosomal karyotyping should be removed from any routine DEE diagnostics protocols and should only be considered for validation analyses as well as for otherwise unresolved cases with remaining specific suspicion of ring chromosomes.

## Conclusions and outlook

According to the ILAE, genetic testing in cases with isolated FE or GE is *per se* not recommended but can be considered in individuals with (i) a positive family history, (ii) additional symptoms (such as intellectual disability, autism, dysmorphism, etc.), and/or (iii) pharmacoresistance [11]. By contrast, genetic testing is highly recommended in individuals with neurodevelopmental disorders with epilepsy, in particular DEE [11].

The method of first choice for genetic testing of epileptic disorders (in particular of DEE) is exome sequencing [11]. It should include analysis of both SNVs and CNVs and should preferably be performed within a trio approach. In cases where exome sequencing failed to deliver a genetic diagnosis, various testing approaches can be considered as second-tier diagnostics, including chromosomal karyotyping, MS-MLPA, and fragment length analysis, depending on the individual symptoms of the affected person [11].

This diagnostic procedure does not only allow for the currently highest diagnostic yield and a reasonably quick turn-around time, it also has been proven to be the most cost-efficient approach [10].

With calculation of polygenic risk scores, RNA sequencing, methylome analysis, long-read sequencing, and many more, yet additional approaches are already on the horizon and may further change our routine diagnostic genetic testing approaches in the not too far away future.

A growing number of genetic epilepsies is associated with precision medicine approaches targeting the molecular pathomechanism. These tailored therapy approaches include e. g. targeted treatment of channelopathies with blockers or agonists, depending on the functional consequence of the respective variant in the affected gene (mutations may for example affect voltage-gated sodium or potassium channels or glutamate or acetylcholine receptors), and dietary interventions (e. g. a ketogenic diet in patients with GLUT1 deficiency or supplementation of vitamin B6 in patients with pyrimidine biosynthesis deficiency) [11].

With several national initiatives on their way aiming for implementation of exome and genome sequencing into routine genetic testing protocols, it appears highly recommendable to reconsider the replacement of step-by-step diagnostics with exome-first (or even genome-first) approaches to allow for saving costs, time, and effort, enabling comprehensive and contemporary genetic testing strategies.
